# The evolution of malignant and reactive γδ + T cell clones in a relapse T-ALL case after allogeneic stem cell transplantation

**DOI:** 10.1186/1476-4598-12-73

**Published:** 2013-07-12

**Authors:** Shaohua Chen, Xin Huang, Haitao Zheng, Suxia Geng, Xiuli Wu, Lijian Yang, Jianyu Weng, Xin Du, Yangqiu Li

**Affiliations:** 1 Key Laboratory for Regenerative Medicine of Ministry of Education, Jinan University, Guangzhou, 510632, China; 2 Institute of Hematology, Medical College, Jinan University, Guangzhou, 510632, China; 3 Department of Hematology, Guangdong General Hospital (Guangdong Academy of Medical Sciences), Guangzhou, 510080, China

**Keywords:** γδ T cell clone, T-ALL, FT-CGH, TCR, Allo-HSCT

## Abstract

**Background:**

To improve the outcome of patients with T-cell acute lymphoblastic leukemia (T-ALL), characterization of the biological features of T-ALL blast cells and the immune status of patients with T-ALL is needed to identify specific therapeutic strategies.

**Findings:**

Using a novel approach based on the combination of fine-tiling comparative genomic hybridization (FT-CGH) and ligation-mediated PCR (LM-PCR), we molecularly identified a malignant γδ + T cell clone with a Vδ5Dδ2Jδ1 rearrangement that was paired with a T cell receptor (TCR) VγI and comprised a Vγ1Vδ5 T cell clone in a relapse T-ALL patient. This malignant Vδ5 T cell clone disappeared after chemotherapy, but the clone was detected again when disease relapsed post allogeneic hematopoietic stem cell transplantation (allo-HSCT) at 100 weeks. Using PCR and GeneScan analyses, the distribution and clonality of the TCR Vγ and Vδ subfamilies were examined before and after allo-HSCT in the patient. A reactive T cell clone with a Vδ4Dδ3Jδ1 rearrangement was identified in all samples taken at different time points (i.e., 4, 8, 68, 100 and 108 weeks after allo-HSCT). The expression of this Vδ4+ T cell clone was higher in the patient during complete remission (CR) post allo-HSCT and at disease relapse.

**Conclusions:**

This study established a sensitive methodology to detect T cell subclones, which may be used to monitor minimal residual disease and immune reconstitution.

## Findings

T-cell acute lymphoblastic leukemia (T-ALL) comprises 25% of adult ALL cases, and its outcome is poorly understood. Among patients with T-ALL, approximately 40% achieve long-term remission [1-3]. Allogeneic hematopoietic stem cell transplantation (allo-HSCT) remains one of the best options for curing T-ALL. However, many patients cannot find an HLA-matched donor; therefore, haploidentical/mismatched HSCTs may be an alternative treatment for T-ALL [4,5]. The high T-ALL failure rate is mainly the result of an insufficient understanding of T-ALL biology, which hampers the identification of reliable prognostic factors that enable appropriate therapy adjustment [1]. To improve T-ALL outcome, characterization of the biological features of T-ALL blast cells and the immune status of patients is needed to design specific therapeutic strategies [6-10]. T-ALL is generally considered to be a clonal disorder that arises from the expansion of committed lymphoid precursors, and leukemic clones in different patients vary due to the T cell receptor (TCR) gene rearrangements that occur during T-cell differentiation [1,11]. Moreover, TCR rearrangements also provide different recombination breakpoints that lead to the creation of fusion genes [12]. TCR rearrangement analysis may be used to determine T-ALL immunogenetic characteristics, and TCR rearrangements may be characterized by leukemia antigen-reactive T cell clones, which are thought to be specific to anti-leukemic cytotoxic T cells [13,14].

In this study, using a novel approach based on the combination of fine-tiling comparative genomic hybridization (FT-CGH) and ligation-mediated PCR (LM-PCR) [15], which combines PCR and the GeneScan techniques [16,17], we molecularly characterized the malignant and reactive γδ + T cell clones in a patient with T-ALL before and after relapse 100 weeks post allo-HSCT.

A 25-year-old male patient was diagnosed with relapse T-ALL in November 2009. The diagnosis was based on cytomorphology, immunohistochemistry and cytoimmunological analyses. He underwent salvage chemotherapies for the next 3 months. However, his response assessments were partial remission (PR), minor remission (NR), and NR. In March 2010, the patient received an HLA-identical sibling peripheral blood (PB) HSCT after a conditioning regimen in addition to cyclosporin (CsA) in combination with a short course of mycophenolate mofetil (MMF) and four doses of methotrexate (MTX) for graft versus host disease (GVHD) prophylaxis as previously described [18]. Lumbar puncture and intrathecal chemotherapy were performed as normal. Four weeks after transplantation, the patient was stricken with fever, diarrhea, and a rash, which is considered Grade II GVHD. Eight weeks after transplantation, the GVHD was controlled with methylprednisolone (MP) and CsA treatment. Central nervous system leukemia (CNSL) was found 20 weeks post transplantation, and intrathecal chemotherapy and radiotherapy of the head were then applied. The patient achieved complete remission (CR) in the bone marrow and was consistently normal upon cerebrospinal fluid examination at 40, 52, and 68 weeks after transplantation. However, relapse was discovered 100 weeks after transplantation, and he underwent chemotherapy over the next two months and achieved remission. The treatment process is summarized in Table 1, and the details of the regimens used are summarized in Additional file 1: Table S1. As of this reporting, the patient remains in follow-up. Blood samples were collected with informed consent when the patient was diagnosed with relapse T-ALL before transplantation and at 4, 8, 68, 100 and 108 weeks post transplantation (Table 2 and Additional file 1: Table S2). PB was collected in an EDTA-containing collection tube, and PB mononuclear cells (PBMCs) were separated using the Ficoll-Hypaque gradient centrifugation method. All procedures were conducted in accordance with the guidelines of the Medical Ethics committees of Guangdong General Hospital according the guidelines of the health bureau of Guangdong Province, China.

**Table 1:** Clinical therapy details for the patient with relapse T-ALL

**Therapy date**	**Therapy**	**Response**	**Blast cells (%) post-treatment**	**CSF**	**Intrathecal chemotherapy**
			**BM / PB**		
18.11-01.12.2009	CTX, VCR, ADM, DXM	PR	11 / 3	-	MTX + DXM
18.12-22.12.2009	MTX, Ara-c , DXM	NR	37 / 3		
25.01-30.01.2010	CTX, Ara-c, TPT	NR	47 / 1	-	Ara-c + DXM
05.03.2010	Allo-HSCT (conditioning regimen: Flu, BU/CY)	CR	1 / 0	-	MTX + DXM
13.04.2010	CsA, MP	GVHD (Grade II)	0.5 / 0		
13.05.2010	CsA, MP	GVHD under control	1.5 / 0	-	MTX + DXM
12.08.2010- 07.01.2011	Radiotherapy	CNSL cured	0 / 0	+	(Ara-c + MTX + DXM) x 8 times
26.04-21.05.2012	VDS, NVT, L-ASPDXM	Relapse, CR	3 / 0	-	MTX + DXM (03.05 2012) Ara-c + DXM (25.05.2012)
08.06-06.07.2012	VCR, NVT, L-ASP, DXM, CTX	CR	1 / 0	-	

Notes: *ADM* adramycin, *Ara-c* cytarabine, *BM* bone marrow, *BU* busulfan, *CNSL* central nervous system leukemia, *CsA* cyclosporin, *CSF* cerebrospinal fluid,*CTX* cyclophosphamide, *CR* complete remission, *CY* cyclophosphamide, *DXM* dexamethasone, *Flu* fludarabine, *L-ASP* l-asparaginase, *MP* methylprednisolone,*MTX* Methotrexate, *NR* minor remission, *NVT* mitoxantrone, *PR* partial remission, *PB* peripheral blood, *TPT* Topotecan, *VCR* vincristine, *VDS* Vindesine.

**Table 2:** Clinical details of the collected samples

**No.**	**Diagnosis**	**Disease status**
A	Relapse	Relapse
B	Pre allo-HSCT	NR
C	4 W post allo-HSCT	CR
D	8 W post allo-HSCT	CR
E	68 W post allo-HSCT	CR
F	100 W	Relapse
G	108 W	MRD

Notes: allo-HSCT: *CR* complete remission,

*MTD* minimal residual disease, *NR* minor remission.

### Malignant T-ALL clone

To characterize the cellular T-ALL features and the T cell clonality at different time points before and after allo-HSCT and at relapse post allo-HSCT, which may identify a factor associated with outcome, we analyzed the TCR breakpoint loci to identify chromosomal translocations and malignant T cell clones by the FT-CGH, LM-PCR, RT-PCT and GeneScan techniques [15-17].

FT-CGH using overlapping oligonucleotides designed to cover an entire genomic region of interest is a valuable tool for high-resolution chromosomal breakpoint characterization [15]. To achieve high resolution CGH (<1 kb), which is necessary for subsequent *in vitro* DNA amplification, a custom designed high-density, fine-tiling long oligonucleotide array consisting of 385,000 oligonucleotides 40–60 bp in length was prepared using Maskless Array Synthesizer (MAS) technology (NimbleGen Systems; Reykjavik, Iceland). This array, covering 24 Mb of genome, was selected using the human genome browser hg18 assembly (University of California, Santa Cruz). The array included TCR αδ and IgH loci, which are located on chromosome 14q11 (Chr14: 21,130 -22,130 kb) and 14q32 (Chr14: 105,080 -106,360 kb), respectively, and known to be frequently involved in chromosomal alterations in lymphoid malignancies. The neighboring oligonucleotides with an average distance of 63 bp were grouped in 200, 400, and 1,000 bp clusters. After normalization with reference DNA from the HEK293 T cell line, the mean fluorescence was analyzed using the SignalMap software (NimbleGen) [15].

**Figure 1:**
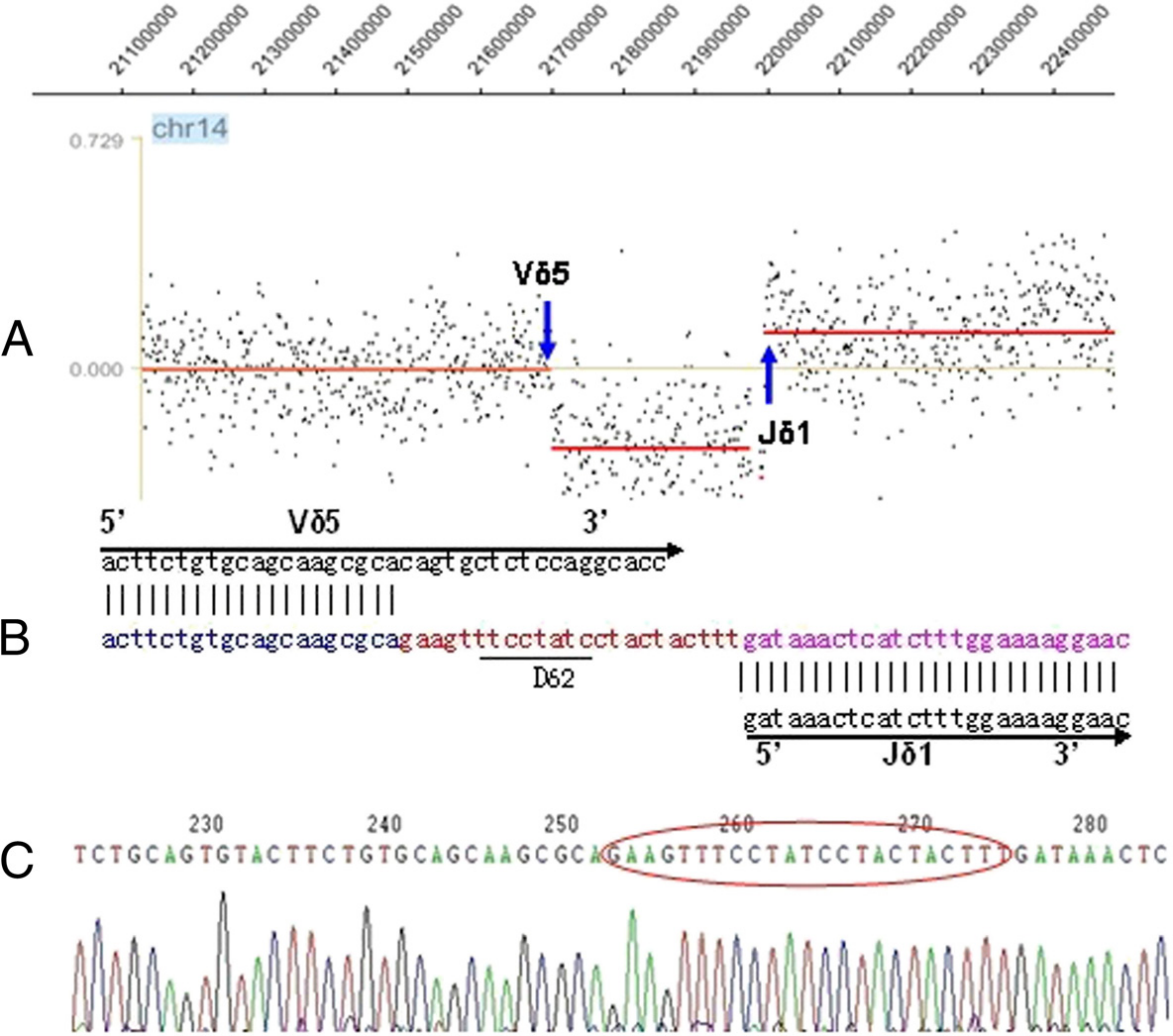
**One TCR δ clone was identified by FT-CGH, LM-PCR and sequencing in a T-ALL case, and a Vδ5Dδ2Jδ1 rearrangement was confirmed. A:** FT-CGH analysis results for the TCR αδ locus in chromosome 14 with arrows indicating the breakpoints. **B:** Comparison of the GenBank sequences within the Vδ5, Dδ2, and Jδ1 segments. **C:** Sequence of the Vδ5Dδ2Jδ1 splice junction from a purified LM-PCR product. The sequences within the ellipse indicate the CDR3 segment including the Dδ2 and N regions.

**Figure 2:**
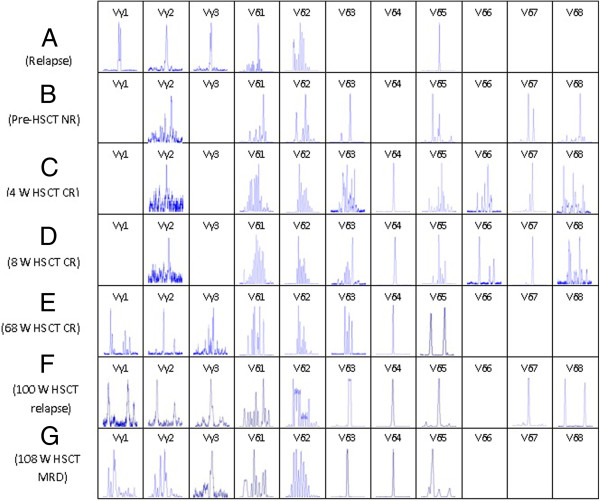
**The distribution and clonality of the TCR V**γ **and V**δ **subfamily T cells in a patient with T-ALL at different time points before and after allo-HSCT. A:** T-ALL relapse, **B:** pre-HSCT, **C:** 4 weeks post allo-HSCT, **D:** 8 weeks post allo-HSCT, **E:** 68 weeks post allo-HSCT, **F:** 100 weeks post allo-HSCT and disease relapse, and **G:** 108 weeks post allo-HSCT and after one cycle of chemotherapy. The monoclonal Vδ5 subfamily could be found in samples **A**, **F**, and **G**, and the oligoclonal Vδ4 subfamily could be identified in samples **C** through **G**.

**Figure 3:**
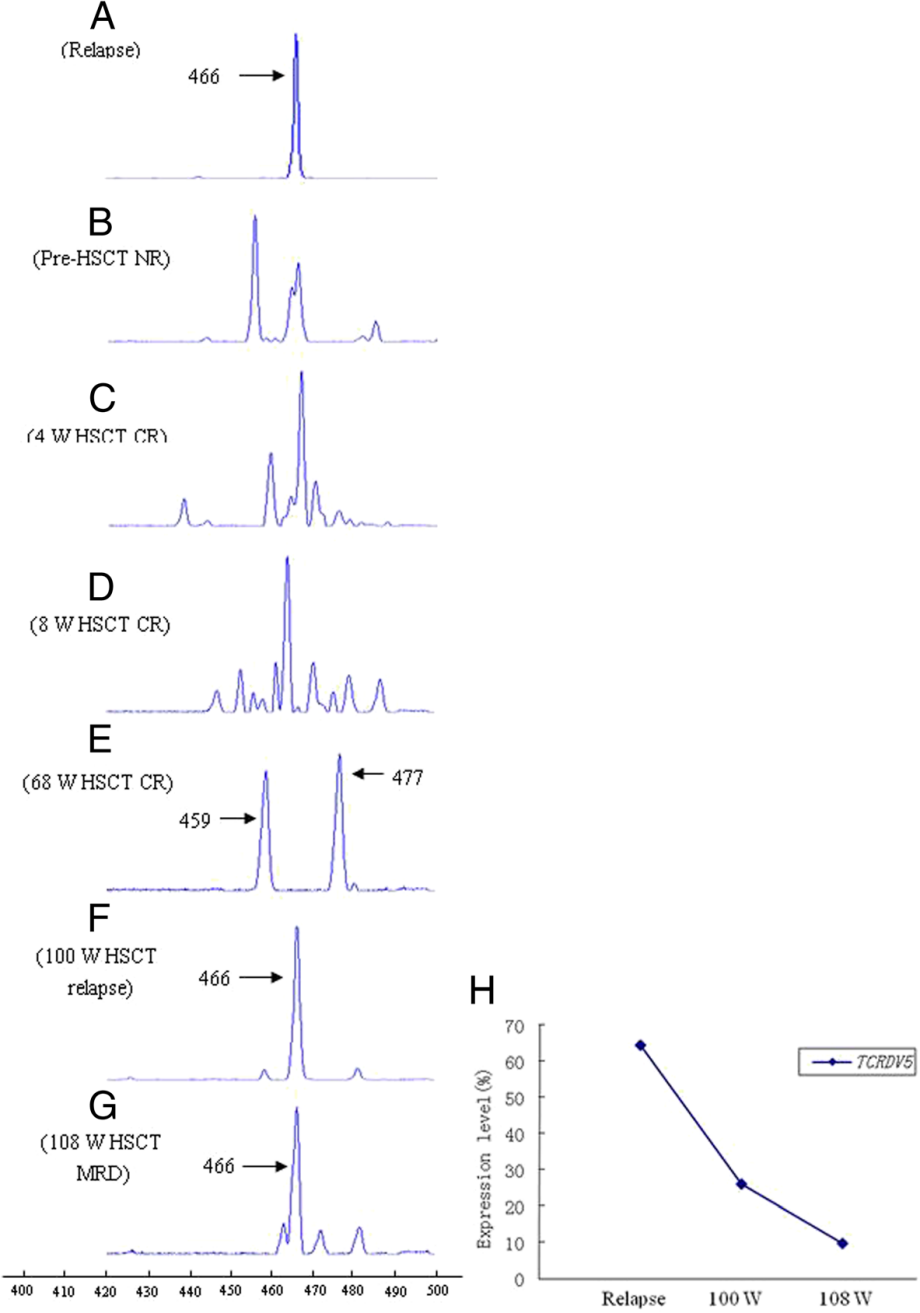
**The clonality and size of Vδ5 T cells in a patient with T-ALL at different time points.** Vδ5 T cell clonality was measured at different time points including the following: **A:** relapse before allo-HSCT (the monoclonal Vδ5 product size was 466 bp), **B:** pre-HSCT, **C:** 4 weeks post allo-HSCT, **D:** 8 weeks post allo-HSCT, **E:** 68 weeks post allo-HSCT (the clonal Vδ5 products were 459 and 477 bp, there were different from the malignant T cell clone with 466 bp), **F:** 100 weeks post allo-HSCT and disease relapse (a 466 bp monoclonal Vδ5 product is again observed), and **G:** 108 weeks post allo-HSCT and after one chemotherapy cycle with minimal residual disease (MRD) remaining (a 466 bp monoclonal Vδ5 product is still detectable). **H:** The expression level of the monoclonal the Vδ5 gene at different time points (i.e., relapse before allo-HSCT, relapse at 100 and 108 weeks post allo-HSCT and after one chemotherapy cycle with MRD remaining).

FT-CGH analysis of the TCR αδ locus (Chr 14: 21,130-22,130) from the T-ALL sample revealed breakpoints at the 21,700 kb (TCR Vδ5) and 22,000 kb (TCR Jδ1) loci (Figure 1A). LM-PCR using nested forward primers specific to the TCR Vδ5 locus revealed a normal rearrangement i.e., a TCR Vδ5 to Jδ1 rearrangement. Direct sequencing of these LM-PCR products demonstrated the following details of the Vδ5Dδ2Jδ1 rearrangement: the Vδ5 breakpoint is located in chromosome 14 at position 21,701,606 and the Jδ1 breakpoint is located in chromosome 14 at position 21,988,926 (Figures 1B and C). To confirm this TCR rearrangement, we used PCR to detect the Vδ5 and Jδ1 loci with specific primers and undigested genomic DNA. Thus, two 14q11.2 (TCR αδ) chromosome breakpoints were characterized as a TCR idiotype rearrangement. Therefore, it may be concluded that there were no additional breakpoints providing a recombination point for the TCR αδ locus-related chromosomal translocation because the translocation of proto-oncogenes to TCR loci most likely occurred as the cell attempted to undergo V(D)J recombination, and it is likely that these translocations arise as a mistake in this process, depending on the location of the breakpoint in the receptor locus [12,15,19,20]. Using RT-PCR, GeneScan, and sequencing analysis, the Vδ5Dδ2Jδ1 rearrangement was confirmed to be monoclonal (The information of the primers used for TCR Vδ5-PCR was listed in Additional file 1: Table S3). Monoclonally expanded Vδ5 T cells, which were presumed to pair with the TCR VγI, comprised a VγIVδ5 T cell clone (Figure 2A). Significantly, this malignant monoclone was lost after chemotherapy and allo-HSCT, but there was a gain in polyclonal Vδ5 T cells, which may pair with VγII T cells and comprise many normal VγIIVδ5 T cell clones (Figure 2B) that arise from normal random V(D)J rearrangements [1]. Although the GeneScan results revealed biclonal Vδ5 T cells in the allo-HSCT sample taken after 68 weeks, these T cell clones were different from the malignant clone (Figure 3). The malignant monoclonal Vδ5 product was 466 bp (Figure 3A), while both of the clonal Vδ5 products from the post transplantation sample were 459 and 477 bp (Figure 3E). Moreover, the malignant monoclonal Vδ5 clone reemerged 100 weeks after allo-HSCT treatment (Figure 3F), a time at which the patient underwent relapse, and the Vδ5 subfamily also included an oligoclone 108 weeks after allo-HSCT (Figure 3G), which was after chemotherapy and when the patient achieved remission. The CDR3 sequence of the Vδ5 clones at diagnosis and 100 and 108 weeks post allo-HSCT were determined, and the polyclonal and biclonal Vδ5 T cell clones were found to contain different CDR3 sequences with different sizes. The Vδ subfamily members preferentially expressed in PB from healthy and T-ALL clones include Vδ1 and Vδ2, while the Vδ5 clone was reported to be rare [21,22]. Thus, this malignant T cell clone may serve as a biomarker for the detection of minimal residual disease. Indeed, a malignant Vδ5 clone containing the same sequence was found at disease relapse 100 weeks post allo-HSCT. Using quantitative real-time PCR [23,24], we found that the expression level of the malignant Vδ5+ T cell clone was high at diagnosis and low when disease relapse was detected. In addition, the Vδ5 expression level declined even further after chemotherapy at 108 weeks when the disease achieved remission with minimal residual disease (i.e., 4% blast cells in the bone marrow) (Figure 3). These data further indicate the significance of dynamically monitoring malignant T cell clones to molecularly identify relapse, which may provide evidence for commencing treatments such as preventive anti-leukemia therapy to clinically inhibit disease relapse.

The identification of malignant T-ALL cell clones was performed at different times using Southern blot, PCR, RT-PCR, GeneScan, FT-CGH, and next generation sequencing spectratyping [25]. The distribution profiles and clonality of the TCR repertoire in T cells could be characterized using RT-PCR and GeneScan [17]. While an advantage of the FT-CGH and LM-PCR techniques is that they can identify chromosomal breakpoints and unique, high percentage T cell clones in a sample, they cannot characterize the polyclonal TCR subfamily distribution or a small fraction of T cell clones [12]. The novel, exhaustive T cell repertoire sequencing technique can directly measure the TCR repertoire size of at least 1 million clonotypes [25]. Therefore, the combination of the FT-CGH, LM-PCR, PCR, and GeneScan techniques for characterizing T cell malignancies is an ideal serial method not only for identifying abnormal chromosome rearrangements in malignant T cell clones but also for detecting the evolution of malignant T cell clones for the diagnosis, prognosis, and evaluation of reactive T cell clones to characterize the immune status of patients and develop specific immunotherapies.

### Clonally expanded reactive T cell clone

**Figure 4:**
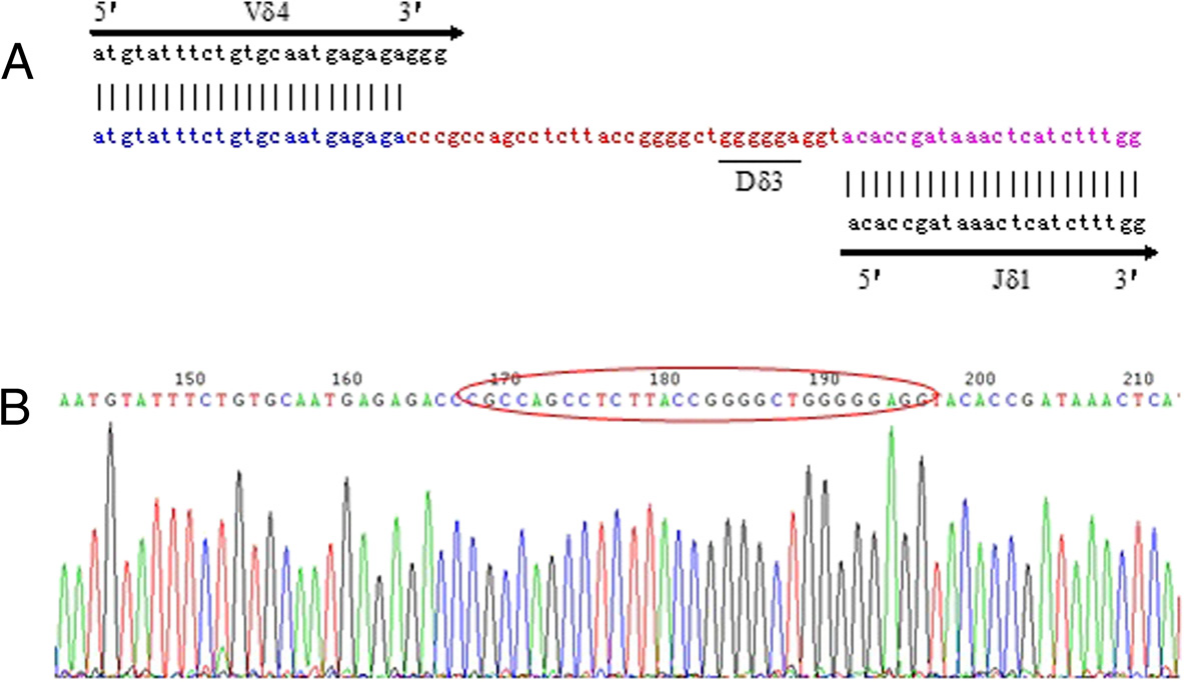
**The CDR3 sequence in the Vδ4Dδ3Jδ1 rearrangement. A:** Sequence comparison of the Vδ4, Dδ3, and Jδ1 segments from GenBank; **B:** sequence of the Vδ4Dδ3Jδ1 splice junction.

**Figure 5:**
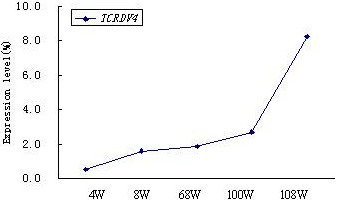
**The Vδ4 gene expression level at different time points post allo-HSCT.**

Numerous studies have demonstrated that specific clonally expanded T cells may be identified in the PB of patients with cancer and leukemia, demonstrating their specific anti-leukemic cytotoxicity in vitro [13,26-28]. Moreover, clonally expanded T cells may be derived from donor lymphocytes after allo-HSCT or donor lymphocyte infusion (DLI), which may increase survival following allo-HSCT in patients with advanced-stage acute leukemia [26,29]. These clonally expanded T cells are derived from TCR αβ or γδ T cells [10]. Increasing data have demonstrated that γδ T cells may be used to develop specific immunotherapies for patients with cancers such as bladder cancer and hepatocellular carcinoma [30]. After transplantation, the patient achieved CR, and the distribution and clonality of the TCR Vγ and Vδ subfamilies were examined. While a malignant Vδ5+ T cell clone was not detected, oligoclonally expanded Vδ4 subfamily T cells were identified in samples from all time points (i.e., 4, 8, 68, 100, and 108 weeks post allo-HSCT) even after the patient underwent relapse (100 weeks post allo-HSCT; Figures 2C to G). The TCR sequence of the Vδ4+ T cell clone was identified as Vδ4Dδ3Jδ1 by direct sequencing, and the same TCR rearrangement was confirmed in all samples from each of the time points post transplantation (Figure 4). The Vδ4+ T cell clone increased more than three-fold when the patient reached CR status post allo-HSCT. Interestingly, the Vδ4 expression level gradually increased 100 and 108 weeks post allo-HSCT, which was when disease relapse occurred and after chemotherapy was given, respectively (Figure 5). This observation suggests that the increased Vδ4+ T cell clone may be a reactively expanded T cell clone that has specific anti-T-ALL function. Although the patient underwent GVHD 8 weeks post transplantation, which was subsequently controlled, the continuously expanded T cell clone may be not related to GVHD. The higher proportion of Vδ4+ T cell clones in samples taken after disease relapse may further support the idea of its role as a reactively expanded anti-leukemia T-cell clone. These results are similar to findings by Meeh et al. who demonstrated that Vδ1+ T cells respond to acute leukemia [28]. However, further characterization of the biological functions of the Vδ4+ T cell clone is needed; the reactive T cell clone may be amplified and used to study adoptive anti-leukemia immunotherapy, moreover, the TCR Vδ4 and its pattner Vγ gene could be used for transfer and the modification of normal T cells for identification their anti-leukemia effect [6,14,31,32].

In summary, the evolution of malignant TCR γδ + and reactive T cell clones was identified in a patient with relapse T-ALL before and after allo-HSCT and at relapse post allo-HSCT. The techniques used in this study establish the sensitive detection of malignant and reactive T cell clones, and the identified T cell clones may serve not only as biomarkers for minimal residual disease detection but also as anti-leukemia immune status indicators in patients who achieved CR.

## Competing interests

The authors declare that they have no competing interests.

## Authors’ contributions

YQL contributed to the concept development and study design. SHC performed the RT-PCR, GeneScan and real-time PCR analyses, HTZ performed LM-PCR, SXG prepared the RNA and cDNA, XLW and LJY prepared the PBMCs and DNA, and XH, JYW and XD were responsible for treatment of the patient and performed clinical data acquisition. YQL, SHC and XH coordinated the study and helped draft the manuscript. All authors read and approved the final manuscript.

## Supplementary Material

Additional file 1: Table S1 Details of Clinical therapy for the patient with relapse T-ALL. **Table S2.** Clinical patient characteristics. **Table S3.** List of primers used for the Vδ5 TCR PCR. Click here for file
