# Supplementary material for: The evolution of malignant and reactive γδ + T cell clones in a relapse T-ALL case after allogeneic stem cell transplantation
Source: Mol Cancer. 2013 Jul 12;12:73. doi: 10.1186/1476-4598-12-73 (PMC3717050; doi:10.1186/1476-4598-12-73)
Supplement: Additional file 1: Table S1 — Details of Clinical therapy for the patient with relapse T-ALL. Table S2. Clinical patient characteristics. Table S3. List of primers used for the Vδ5 TCR PCR. [file 1476-4598-12-73-S1.doc]

Additional files

**Table: S1. Details of Clinical therapy for the patient with relapse T-ALL**

| Therapy Date | Therapy | Blood routine of pre-treatment | | | Response | Blast cells (%)  post-treatment | CSF | Intrathecal chemotherapy |
| --- | --- | --- | --- | --- | --- | --- | --- | --- |
|  |  | WBC  (10^9^/L) | Hb(g/L) | PLT  (10^9^/L) |  | BM / PB |  |  |
| 18.11-01.12.2009 | CTX (450mg, d1-d3)  VCR( 2mg, d4,d11)  ADM (80mg, d4)  DXM(40mg,d1-d4, d11-14) | 18.74 | 135.3 | 145.6 | PR | 11 / 3 | - | MTX15mg+DXM5mg |
| 18.12-22.12.2009 | MTX (1.6g, d1 )  Ara-c (4.5g, d2-d3)  DXM(10mg, d2-d3) | 3.39 | 102.5 | 391.8 | NR | 37 / 3 |  |  |
| 25.01-30.01.2010 | CTX (0.5g, d1-d3)  Ara-c(1.6g,d2-d6)  TPT(2mg, d2-d6) | 7.8 | 106 | 676 | NR | 47 / 1 | - | Ara-c50mg+DXM5mg |
| 05.03.2010 | Allo-HSCT (conditioning regimen: fludarabine, BU/CY) | 4.97 | 109.9 | 459.5 | CR | 1 / 0 | - | MTX15mg+DXM5mg |
| 13.04.2010 | MP(80mg,qd)  CsA (50mg, q12h) | 7.03 | 87 | 16 | GVHD (Grade II) | 0.5 / 0 |  |  |
| 13.05.2010 | [MP](app:ds:prednison)(50mg, Po.qd)  CsA(90mg, Po.qd) | 5.84 | 110 | 117 | GVHD under control | 1.5 / 0 | - | MTX15mg+DXM5mg |
| 12.08.2010-07.01.2011 | Intrathecal chemotherapy and radiotherapy  (DT: 24Gy x 12 times) | 6.75 | 124 | 149 | CNSL cured | 0 / 0 | + | (Ara-c50mg+MTX15mg+DXM5mg) x 8 times |
| 26.04-21.05.2012 | VDS(5mg, d1,8,15,22)  NVT (15mg, d1-3)  L-ASP(10000U, d11,14,17,20,23)  DXM(10mg, 4d/w x 4weeks) | 8.24 | 147.1 | 265.4 | Relapse - CR | 3 / 0 | - | (MTX10mg+DXM5mg,03.05 2012)  (Ara-c40mg+DXM5mg,25.05.2012) |
| 08.06-06.07.2012 | VCR ( 2mg, d1,8,15,22)  NVT (10mg, d1-3;15mg d4)  L-ASP (10000U, d11,14,17,20,23,26)  DXM (10mg, 4d/w x 4weeks)  CTX (1.2g, d1,15) | 6.94 | 91 | 332 | CR | 1 / 0 | **-** |  |

Notes: ADM: adramycin, Ara-c: cytarabine, BM: bone marrow, BU: busulfan, CNSL: central nervous system leukemia, CsA: cyclosporin, CSF: cerebrospinal fluid, CTX: cyclophosphamide, CR: complete remission, CY: cyclophosphamide, DXM: dexamethasone, Flu: fludarabine, L-ASP: l-asparaginase, MP: methylprednisolone, MTX: Methotrexate, NR: minor remission, NVT: mitoxantrone, PR: partial remission, PB: peripheral blood, TPT: Topotecan, VCR: vincristine, VDS: Vindesine

**Table: S2. Clinical patient characteristics**

| No | Date | Diagnosis | Blast (%) | | Blood routine | | | Cytoimmunological analysis |
| --- | --- | --- | --- | --- | --- | --- | --- | --- |
|  |  |  | BM | PB | WBC  (10^9^/L) | Hb  (g/L) | PLT  (10^9^/L) |  |
| A | 2009-11-16 | relapse | 75 | 62 | 18.74 | 135.3 | 145.6 | 89% positive for CD71, CD10, CD7, CD13, CD56, TdT, cytoCD3, and CD34(part) |
| B | 2010-2-25 | Pre allo-HSCT | 47 | 1 | 4.97 | 109.9 | 459.5 | 27.73% positive for CD71, CD10, CD7, CD13, CD56, TdT, cytoCD3, and CD34(part) |
| C | 2010-4-13 | 4 W post allo-HSCT | 1 | 0 | 7.03 | 87 | 16 | MRD: 0.10% |
| D | 2010-5-13 | 8 W post allo-HSCT | 2 | 0 | 5.84 | 110 | 117 | MRD: undetectable |
| E | 2011-8-31 | 68 W post allo-HSCT | 2 | 0 | 8.55 | 158 | 211 | MRD: undetectable |
| F | 2012-4-10 | 100 W | 33.5 | 1 | 8.24 | 147.1 | 265.4 | MRD：ND |
| G | 2012-6-7 | 108 W | 4 | 0 | 6.94 | 91 | 332 | MRD：1.63％ |

ND: not detected; MRD: minimal residual disease

Table: S3 List of primers used for TCR Vδ5-PCR

| Name | Location | Sequence |
| --- | --- | --- |
| Vδ5-1 | Chr14: 21701126-21701146 | 5’-TCAGCCAGACTGTGAGTTGTG |
| Vδ5-2 | Chr14: 21701300-21701320 | 5’-GGGGTAAACAGTCAACAGAAG |
| Jδ1-1 | Chr14: 21989003-21989022 | 5’-GATGGAGGATGCCTTAACCT |
| Jδ1-2 | Chr14: 21988962-21988982 | 5’-GAGTTACTTACTTGGTTCCAC |
